# Metatranscriptomic characterization of the canine fecal virome from pooled samples in Gansu, China

**DOI:** 10.1016/j.virusres.2025.199666

**Published:** 2025-11-19

**Authors:** Wenhua Gao, Yanhong Yao, Yabo Sun, Wenjing Pu, Lin Xu

**Affiliations:** a Basic Medical School, Gansu Medical College, Pingliang 744000, China; b School of Public Health, Shandong First Medical University and Shandong Academy of Medical Sciences, Ji’nan 250017, China; c Key Laboratory of Emerging Infectious Diseases in Universities of Shandong, Shandong First Medical University and Shandong Academy of Medical Sciences, Ji’nan 250017, China

**Keywords:** Canine virome, Metatranscriptomics, Pooled sampling, Viral zoonoses, Enteric viruses

## Abstract

As popular companion animals, dogs present a potential risk for zoonotic viral transmission through close contact with humans. To characterize the fecal virome of dogs in Pingliang City, Gansu Province, China, we performed metatranscriptomic sequencing on 30 fecal samples pooled into three libraries, representing three distinct living environments. A total of 112,900,200 clean reads were obtained, revealing 16 viral genera spanning 15 families and highlighting a diverse viral community comprising animal viruses, bacteriophages, and plant viruses. Notably, we identified five known pathogenic viruses: canine astrovirus (3708 reads), canine dicipivirus (6578 reads), canine norovirus (16 reads), canine vesivirus (74 reads), and canine rotavirus (128 reads). Their presence suggests possible exposure events at the human-animal interface, although the infectivity and transmission risk require further experimental validation. These findings significantly expand our understanding of the canine virome and underscore the importance of “One Health” surveillance in companion animals. However, the actual zoonotic potential of the detected viruses, remains to be elucidated through further targeted investigation.

## Highlights

• Presents the first comprehensive meta-transcriptomic analysis of the fecal virome in dogs from Pingliang City, China.
• Identifies 16 viral genera (spanning 15 families), comprising animal viruses (19.49 % of viral reads), bacteriophages (0.73 %), and plant viruses (79.78 %), illustrating a complex viral ecosystem within canine hosts.
• Detects five pathogenic viruses—canine astrovirus, canine dicipivirus, canine norovirus, canine vesivirus, and canine rotavirus—highlighting associated zoonotic and animal health concerns.
• Delivers essential baseline data for monitoring viral zoonoses and underscores the importance of strengthened surveillance at the human-animal interface.

Viral diseases significantly threaten human and animal health, with animals serving as reservoirs or intermediate hosts (Zhang and Holmes, 2020; Aditya et al., 2021). Companion animals, particularly dogs, share unique bonds with humans. While contact may confer immunological or emotional benefits (Nermes et al., 2015), it also facilitates bidirectional disease transmission. Dogs - the most common companion animals - are established reservoirs for zoonotic viruses, such as rabies virus, influenza virus, monkeypox virus, and norovirus, posing shared health risks (Stull et al., 2015; Borland et al., 2020; Seang et al., 2022; Kumar et al., 2023; Wu et al., 2024).

Metatranscriptomics analysis, by capturing actively transcribed RNA, enables precise taxonomic profiling and quantification of microbial communities (Huang et al., 2022; Shi et al., 2022). This approach has revolutionized viral discovery, revealing unprecedented phylogenetic diversity, genome evolution, and host-virus interactions (Shi et al., 2016, 2018; Zhang et al., 2018; He et al., 2022; C. Chen et al., 2023; Xu et al. 2024). It is also critical for diagnosing emerging viruses (e.g., SARS-CoV-2 genome characterization during COVID-19 (Wu et al., 2020)).

Although recent studies profiled enteric viromes of companion animals in eastern China (Wu et al., 2024), data from western China remains scarce. To bridge this gap, we conducted the metatranscriptomics analysis of the fecal virome in dogs from Pingliang City, Gansu Province, and characterized five known pathogenic viruses, providing foundational data for early diagnosis and prevention of potential zoonotic infections.

**Fig. 1:**
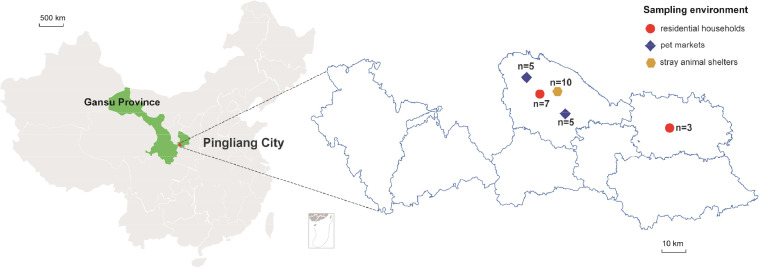
Sampling locations of freshly voided fecal samples from dogs in Pingliang City, Gansu Province, China.

**Fig. 2:**
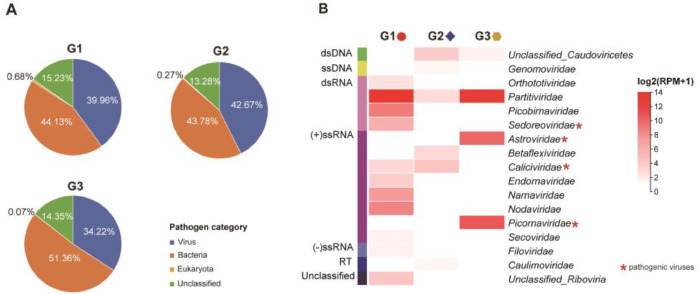
Contig annotation of metatranscriptomic sequencing and diversity of the canine fecal virome. (A) Contig assembly and annotation of metatranscriptomic libraries (G1 - G3). (B) Virus diversity and abundance calculated as log2(RPM+1). Each metatranscriptomic library is labeled according to its sampling environment, as in Fig. 1.

Metatranscriptomic sequencing of three libraries constructed from 30 fecal samples representing three distinct living environments (Fig. 1, Supplementary Table 1) yielded 121,650,439 raw reads. After quality control (filtering the raw data, checking the sequencing error rate, and checking the base quality values), 112,900,200 clean reads were retained across three libraries: G1 (27,021,946), G2 (51,261,806), and G3 (34,616,448) (Supplementary Table 2). *De novo* assembly generated 50,038 contigs, which were then classified via BLAST against NCBI nr/nt databases. Consistent with prior studies in eastern China (Wu et al., 2024), bacterial contigs was a major component of the canine fecal microbiome (24,195, accounted for 48.35 % of the clean reads), followed by viral contigs (18,694, 37.36 %), unclassified contigs (7055, 14.10 %), and eukaryote contigs (94, 0.19 %). The low eukaryotic representation (0.19 %) aligns with limited fungal biomass in healthy canine guts (Mukherjee et al., 2015). Viral contigs represented 34.22–42.67 % of classified sequences per library, while bacterial contigs accounted for 43.78–51.36 % (Fig. 2A).

Sixteen viral genera (15 families) were identified across all libraries. This expands known canine viral diversity and provides critical data for monitoring emerging pathogens at the human-animal interface. Animal viruses (19.49 % of viral reads) and bacteriophages (0.73 %) were detected at levels consistent with the gut environment. In stark contrast, plant viruses constituted the vast majority of sequences (79.78 %), potentially reflecting dietary intake—a hypothesis that requires future validation. RNA viruses constituted 86.66 % of taxa (four families of dsRNA viruses, eight families of (+)ssRNA viruses, and one families of (-)ssRNA viruses), DNA viruses 6.67 % (one family of ssDNA viruses), and reverse-transcribing viruses 6.67 % (one family). Library-specific profiles revealed that G1-G3 contained ten, five, and three viral families, respectively, among which *Partitiviridae* presence in all of them. To quantitatively assess the composition of the canine fecal virome and rigorously validate key findings, we performed stringent read mapping and coverage analysis. The relative abundance (RPM) of all 15 detected viral families was quantified, and confirmed the distinct virome structure across the three living environments (Fig. 2B).

Library G1 harbored the highest proportions of *Partitiviridae* (RPM = 12,332.75), *Picobirnaviridae* (491.19), and *Nodaviridae* (367.67). Library G2 was defined by a notable presence of *Caliciviridae* (18.89), and Library G3 comprised substantial levels of *Partitiviridae* (6335.48), *Picornaviridae* (1689.29), and *Astroviridae* (952.25) (Supplementary Table 3).

Notably, five pathogenic viruses were detected: canine norovirus (in library G1, RPM=7.72), canine rotavirus (G1, 61.76), canine vesivirus (G2, 18.89), canine astrovirus (G3, 952.25), and canine dicipivirus (G3, 1689.29) (Fig. 2B, Supplementary Table 3). Dogs are reservoirs for zoonoses like rabies and noroviruses (Stull et al., 2015; Borland et al., 2020; Charoenkul et al., 2020; Kumar et al., 2023). Our detection of norovirus underscores cross-species transmission potential. Similarly, canine rotavirus - detected here in dogs but typically human or porcine (Li et al., 2022; Wang et al., 2024) - highlights the role of companion animals in bridging wildlife-livestock-human pathogen flow. These observations reinforce the need for “One Health” surveillance targeting canine-specific pathogens with zoonotic propensity. Canine astrovirus and canine dicipivirus exhibit notably high abundance in G3 constructed from shelter dogs’ fecal samples, signals infection risks in congregate settings.

**Fig. 3:**
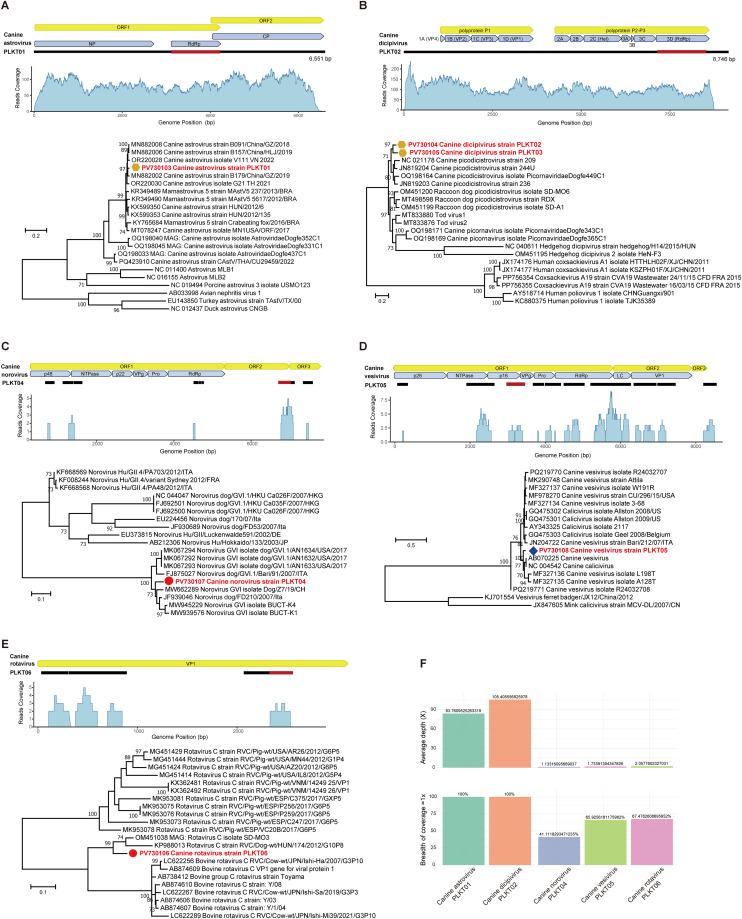
Analysis of contig distribution, coverage, and phylogenetic relationships of identified viruses. The phylogenies were inferred using nt sequences of key functional genes: RdRp gene of (A) *Astroviridae*, and (B) *Picornaviridae*, (C) ORF2 gene of *Norovirus*, and (D) ORF1 gene of *Vesivirus* in the family *Caliciviridae*, and (E) VP1 gene of *Rotavirus C* in the genus *Rotavirus*, family *Sedoreoviridae*, The viral sequences obtain in this study were labeled to match the sampling environments of the detected library, as shown in Fig. 1. Bootstrap support values from 1000 replicates are shown at the nodes. Only values ≥ 70 % are shown. (F) Genome coverage statistics of assembled viral contigs, the top panel shows the average sequencing depth (X) for each virus, while the bottom panel displays the breadth of coverage (percentage of genome covered at ≥1x depth); coverage metrics were calculated from the mapping of RNA-seq reads to the assembled viral genomes, with values presented as mean coverage depth and coverage breadth percentages.

Whole genomes were obtained for canine astrovirus (strain PLKT01, 6551 bp) and canine dicipivirus (PLKT02, 8746 bp), while multiple partial sequences were uncovered for canine norovirus (PLKT04), canine vesivirus (PLKT05), and canine rotavirus (PLKT06). In-depth validation analysis confirmed their presence with robust genome coverage metrics, including read counts, depth of coverage, and breadth of coverage (Fig. 3 and Supplementary Table 3).

Genetic analyses revealed substantial genetic divergence in novel strains, suggesting potential new genotypes within established taxa: canine astrovirus strain PLKT01 shared <94.85 % nt identity with known canine astroviruses; canine dicipivirus strains PLKT02 and PLKT03 showed 95 % nt identity to each other and <88.75 % nt identity to known dicipivirus strains; ORF2 of canine norovirus strain PLKT04 shared <93.81 % nt identity to known norovirus strains; canine vesivirus strain shared <94.41 % nt identity to known canine vesiviruses; the VP1 segment of canine rotavirus strain PLKT06 showed <90.48 % nt identity to known rotavirus strains (Table 1). To determine the evolutionary relationships among the newly identified and previously recognized viruses, phylogenetic trees were generated based on nt sequences of key functional genes using Maximum-likelihood method, and all five newly identified pathogenic viruses were most closely related to relevant viruses from canine samples (Fig. 3). Canine astrovirus strain PLKT01 was closely related to the canine astrovirus strain B179/China/GZ/2019 (Accession No.: MN882002), which was detected in cloacal swabs of Chinese canine (Fig. 3A). Canine dicipivirus strains PLKT02 and PLKT03 were most closely related to canine dicipivirus strains (Accession Nos.: NC_021178, JN819204, OQ198164, JN819203) detected in Chinese canines (Fig. 3B). Canine norovirus strain PLKT04 was closely related to norovirus strain dog/FD210/2007/Ita (Accession No.: JF939046) detected in United Kingdom dog feces (Fig. 3C). Canine vesivirus strain PLKT05 was closely related to canine vesivirus strains (Accession Nos.: NC004542, AB070225) detected in Japanese canines (Fig. 3D). While canine rotavirus strain PLKT06 was closely related to rotavirus C isolate SD-MO3 (Accession No.: OM451038) detected in Chinese raccoon dog (Fig. 3E).

**Table 1:** Pathogenic viruses detected in the present study.

Classification	Virus	Whole genome (bp)	Closest relative (% nt identity)	No.^a^	Positive library	Sampling environment
*Astroviridae*						
*Mamastrovirus*	Canine astrovirus strain PLKT01	6551	Canine astrovirus strain B091/China/GZ/2018 (94.85)	1 (1)	G3	Stray animal shelters
*Picornaviridae*						
*Dicipivirus*	Canine dicipivirus strains PLKT02/PLKT03	8746	Canine picodicistrovirus strain 244 U (88.30/88.75)	2 (1)	G3	Stray animal shelters
*Caliciviridae*						
*Norovirus*	Canine norovirus strain PLKT04	-	Norovirus dog/FD210/2007/Ita (93.81)	1 (0)	G1	Residential households
*Vesivirus*	Canine vesivirus strain PLKT05	-	Canine vesivirus (94.41)	1 (0)	G2	Pet markets
*Sedoreoviridae*						
*Rotavirus*	Canine rotavirus strain PLKT06	-	Rotavirus C isolate SD-MO3 (90.48)	1 (0)	G1	Residential households

a Number of virus strains, with the number in parentheses indicating the whole genomes assembled *de novo* using the Trinity program v2.5.1.

In conclusion, this study used metatranscriptomic sequencing to characterize the fecal virome of dogs from three distinct living environments in Pingliang City, China. Our analyses revealed a diverse viral community comprising animal viruses, bacteriophages, and plant viruses. Notably, we detected sequences of five known pathogenic viruses: canine astrovirus, canine dicipivirus, canine norovirus, canine vesivirus, and canine rotavirus. Critically, the incorporation of metadata provided essential context for interpreting these viromic profiles. The elevated signals of enteric viruses such as canine astrovirus and canine dicipivirus were primarily associated with high-density housing conditions and the presence of symptomatic animals, particularly in the shelter environment, suggesting facilitated fecal-oral transmission. While the infectivity and zoonotic transmission risk of these viruses remain to be experimentally validated, their detection of viruses in closely co-habiting animals underscores possible exposure events at the human-animal interface. Together, these findings significantly expand our understanding of the canine fecal virome and reinforce the importance of “One Health”–driven surveillance in companion animals. Future targeted studies are warranted to clarify the actual zoonotic potential of these viruses.

## 1 Methodology

### 1.1 Samples collection and metadata acquisition

Between December 2023 and May 2024, we collected 30 freshly voided fecal samples from dogs in Pingliang City, Gansu Province, China. The samples represented three distinct living environments: residential households (*n* = 10), pet markets (*n* = 10), and stray animal shelters (*n* = 10) (Fig. 1). For all samples, we systematically recorded metadata pertaining to the sampling site and individual dog characteristics (e.g., age, sex, clinical signs, and health management history), with availability varying considerably across the cohorts (see Supplementary Table 1 for a summary). All samples were immediately frozen in liquid nitrogen upon collection, transported to the laboratory, and maintained at −80 °C until processing.

### 1.2 RNA library construction and sequencing

Fecal samples were pooled (ten individuals per pool, see Supplementary Table 2 for pooling strategy) based on the collection environment, and total RNA was extracted using RNeasy Plus Universal Mini Kit (Qiagen). RNA integrity and quantity were measured using the RNA Nano 6000 Assay Kit of the Bioanalyzer 2100 system (Agilent Technologies). Then the libraries were constructed following the TruSeq stranded total RNA paired-end libraries protocol (Illumina). The library preparation and sequencing were conducted by our commercial partner, Novogene Co., Ltd., following their standardized high-throughput workflows. Total RNA was treated with DNase I to eliminate genomic DNA contamination, and the ribosomal RNA (rRNA) was depleted using the Ribo-Zero Plus Kit (Illumina). 500 ng RNA was then fragmented into 250-300 bp fragments and reverse-transcribed into cDNA subsequently. Remaining overhangs of double-strand cDNA were converted into blunt ends via exonuclease/ polymerase activities. After adenylation of 3′ ends of DNA fragments, sequencing adaptors were ligated to the cDNA. In order to select cDNA fragments of preferentially 250-300 bp in length, the library fragments were purified with AMPure XP system (Beverly). Amplification was performed by polymerase chain reaction (PCR) with Phusion High-Fidelity DNA polymerase (NEB), Universal PCR primers and Index (X) Primer. Library quality was measured by the Qubit fluorometer (Thermo Fisher) and adjusted to 1 ng/μL. Agilent 2100 Bioanalyzer was deployed to examine the insert size of the acquired library. At last, the accurate concentration of cDNA library was again examined using qPCR. Once the insert size and concentration (maintained above 2 nM) of the library was identical, the samples can then be subjected for sequencing. To rule out cross-talk and laboratory contamination, negative controls were included in the same library preparation batch. Paired-end (150 bp) sequencing of each RNA library was performed on the Nova X-plus platform (Illumina). DNA viruses were detected via RNA libraries.

### 1.3 Transcriptome analysis and virus discovery

Bioinformatic analyses of the sequencing reads were undertaken as described previously (Shi et al., 2015). Briefly, raw reads were quality-controlled and preprocessed with the Fastp programme v0.20.0 (Chen et al., 2018). Eukaryotic rRNAs were removed with Bowtie2 v2.3.3.1 (Langmead et al., 2012), and the resultant reads were *de novo* assembled using Trinity program v2.5.1 (Grabherr et al., 2011) under default parameters. The assembled contigs were identified by querying to the NCBI non-redundant nucleotide (nt) and protein (nr) database using Blastn and Diamond respectively, with e-values both set to 1E-5. To balance specificity with sensitivity for novel virus discovery, candidate viral sequences were subsequently subjected to manual curation and re-Blast analysis to confirm viral origin. The transcripts obtained from Trinity were quantified using the RSEM program (Li et al., 2010). To validate viral discoveries, clean reads were mapped to reference genomes of all detected viral families using Bowtie 2 (v2.5.1). The mapping results were normalized as Reads Per Million (RPM) for cross-sample comparison. The viral abundance of various families in each transcriptome was plotted using TBtools (Y.M. Chen et al., 2023). For the concerned viruses, the breadth of coverage (percentage of genome covered ≥1x) and average depth of coverage were calculated using SAMtools (v1.17) and BEDTools (v2.31.0). Genome coverage plots were generated to visualize read distribution.

### 1.4 Phylogenetic analysis

The Trinity-assembled viral sequences were further processed using the Lasergene software package v5.0 (DNASTAR). Specifically, SeqMan was used for re-assembly, and MegAlign was used for nucleotide sequence identity analysis. Viral sequences were aligned using MAFFT v7 (E-INS-i algorithm) (Katoh and Standley, 2013). Poorly aligned regions were trimmed with TrimAl v1.4.1 (Capella-Gutiérrez et al., 2009). For the sequences used in tree construction, the alignments length and the percentage of gaps were *Astroviridae* (1703 bp, 7.81 %), *Picornaviridae* (2799 bp, 12.83 %), *Norovirus* (480 bp, 15.83 %), *Vesivirus* (482 bp, 3.73 %), and *Rotavirus C* (507 bp, 1.97 %). Best-fit nucleotide substitution models were selected in MEGA v7.0 (Kumar et al., 2016) via Bayesian Information Criterion (BIC): *Astroviridae* and *Picornaviridae* (GTR+G), *Norovirus* (K2+G), *Vesivirus* (K2+G), and *Rotavirus C* (HKY+G+I). Maximum-likelihood phylogenies were inferred using MEGA v7.0 with 1000 bootstrap replicates (Kumar et al., 2016).

## Ethical statement

The animal study protocol was approved by the Gansu Medical College Animal Ethics Committee (Protocol No GSMC20231001). Verbal informed consent was appropriately obtained from all relevant personnel, including pet owners, market operators, and shelter management. All institutional and national guidelines for the care and use of animals were followed.

## CRediT authorship contribution statement

**Wenhua Gao:** Writing – review & editing, Writing – original draft, Visualization, Validation, Resources, Methodology, Funding acquisition, Formal analysis, Conceptualization. **Yanhong Yao:** Writing – review & editing, Validation. **Yabo Sun:** Validation. **Wenjing Pu:** Writing – review & editing, Validation, Supervision. **Lin Xu:** Writing – review & editing, Validation, Supervision, Software, Resources, Methodology.

## Declaration of competing interest

The authors declare that they have no known competing financial interests or personal relationships that could have appeared to influence the work reported in this paper.

## Data Availability

All sequence reads have been deposited in the Short Read Archive BioProject PRJNA1282894. Viral genomes have been submitted to GenBank with Accession Nos PV730103-PV730108.

## Appendix Supplementary materials

Image, application 1

Image, application 2

Image, application 3
