# Supplementary material for: Metatranscriptomic characterization of the canine fecal virome from pooled samples in Gansu, China
Source: Virus Res. 2025 Nov 19;362:199666. doi: 10.1016/j.virusres.2025.199666 (PMC12689224; doi:10.1016/j.virusres.2025.199666)
Supplement: Supplementary file 1 [file mmc1.docx]

**Supplementary Table 1.** Metadata and characteristics of the canine samples included in the present study.

| **Parameter** | **Residential households (N=10)** | **Pet markets (N=10)** | **Stray animal shelters (N=10)** |
| --- | --- | --- | --- |
| **Sampling site characteristics** |  |  |  |
| **Animal source** | Single household | Multiple vendors | Stray intake |
| **Housing density** | Low (Individual) | High (Caged, high turnover) | High (Kenneled, stable) |
| **Health management** | High (Regular veterinary care) | Variable / Low | Medium (Basic care provided) |
| **Animal demographics (availability)** |  |  |  |
| **Age** | High (All known) | Low (Mostly unknown) | Medium (Partially known/estimated) |
| **Sex** | Balanced ratio, some neutered (All known) | Unbalanced ratio (Mostly known) | Mixed, low neuter rate (Partially known/estimated) |
| **Health & Management (Availability)** |  |  |  |
| **Vaccination Status** | Majority complete (All known) | Mostly incomplete / Unknown | Mostly incomplete / Unknown (Partially known/estimated) |
| **Clinical Signs at Sampling** | Majority asymptomatic (All known) | Majority asymptomatic (All known) | Mixed (Asymptomatic, diarrhea, lethargy) (Partially known) |
| **Deworming History** | High (All known) | Medium (Partially known) | Medium (Partially known) |
| **Antibiotic Use (Recent)** | High (All known) | Low (Mostly unknown) | Low (Mostly unknown) |
| **Diet** | Diverse: Kibble, raw, leftovers, home-cooked (All known) | Uniform commercial kibble (All known) | Diverse: Kibble, food scraps, raw, leftovers, Home-cooked (Partially known/estimated) |
