# Supplementary material for: Metatranscriptomic characterization of the canine fecal virome from pooled samples in Gansu, China
Source: Virus Res. 2025 Nov 19;362:199666. doi: 10.1016/j.virusres.2025.199666 (PMC12689224; doi:10.1016/j.virusres.2025.199666)
Supplement: Supplementary file 2 [file mmc2.docx]

**Supplementary Table 2.** Information of RNA library construction and metatranscriptomic sequencing.

| **Library ID** | **Sample No.** | **Sampling environment** | **Raw reads counts** | **Clean reads counts** | **No-rRNA reads** | **Q20 (%)** | **Q30 (%)** |
| --- | --- | --- | --- | --- | --- | --- | --- |
| G1 | 10 | residential households | 34,041,158 | 27,021,946 | 2,072,531 | 95.03 | 89.93 |
| G2 | 10 | pet markets | 52,479,104 | 51,261,806 | 3,917,088 | 97.99 | 94.33 |
| G3 | 10 | stray animal shelters | 35,130,177 | 34,616,448 | 3,893,946 | 98.92 | 96.89 |
