# Supplementary material for: Metatranscriptomic characterization of the canine fecal virome from pooled samples in Gansu, China
Source: Virus Res. 2025 Nov 19;362:199666. doi: 10.1016/j.virusres.2025.199666 (PMC12689224; doi:10.1016/j.virusres.2025.199666)
Supplement: Supplementary file 3 [file mmc3.docx]

**Supplementary Table 3.** The canine fecal virome by metatranscriptomic sequencing

| **Library ID** | **Classification** | | **Read counts** | **RPM** |
| --- | --- | --- | --- | --- |
| G1 | dsRNA | *Partitiviridae*_*unclassified* | 25,560 | 12,332.75 |
|  |  | *Picobirnaviridae*_*Orthopicobirnavirus* | 1,018 | 491.19 |
|  |  | *Sedoreoviridae*_*rotavirus** | 128 | 61.76 |
|  |  | *Orthototiviridae*_*unclassified* | 8 | 3.86 |
|  | ssRNA (+) | *Nodaviridae*_*unclassified* | 762 | 367.67 |
|  |  | *Narnaviridae*_*Mitovirus* | 396 | 191.07 |
|  |  | *Endornaviridae*_*Alphaendornavirus* | 24 | 11.58 |
|  |  | *Caliciviridae*_*Norovirus** | 16 | 7.72 |
|  |  | *Secoviridae*_*Fabavirus* | 2 | 0.97 |
|  | ssRNA (-) | *Filoviridae*_*Marburgvirus* | 2 | 0.97 |
|  | Unclassified | *unclassified_Riboviria* | 38 | 18.34 |
| G2 | dsDNA | *Unclassified_Caudoviricetes* | 50 | 12.76 |
|  | ssDNA | *Genomoviridae_Gemykrogvirus* | 2 | 0.51 |
|  | dsRNA | *Partitiviridae_unclassified* | 22 | 5.62 |
|  | ssRNA (+) | *Caliciviridae_Vesivirus** | 74 | 18.89 |
|  |  | *Betaflexiviridae_Carlavirus* | 26 | 6.64 |
|  | RT | *Caulimoviridae_Tungrovirus* | 2 | 0.51 |
| G3 | dsDNA | *Unclassified_Caudoviricetes* | 4 | 1.03 |
|  | dsRNA | *Partitiviridae_unclassified* | 24,670 | 6,335.48 |
|  | ssRNA (+) | *Picornaviridae_Dicipivirus** | 6,578 | 1,689.29 |
|  |  | *Astroviridae_Mamastrovirus** | 3,708 | 952.25 |

*** Pathogenic viruses detected in the present study.
